# The Foundations of Autistic Flourishing

**DOI:** 10.1007/s11920-023-01441-9

**Published:** 2023-08-08

**Authors:** Elizabeth Pellicano, Melanie Heyworth

**Affiliations:** 1 Department of Clinical, Educational and Health Psychology, University College London, 26 Bedford Way, London, WC1H 0DS UK; 2 Macquarie School of Education, Macquarie University, 29 Wally’s Walk, Sydney, Australia; 3 Reframing Autism, Warners Bay, NSW, Australia

**Keywords:** Ethics, Medical model, Neurodiversity, Autonomy

## Abstract

**Purpose of Review:**

All people—including Autistic people—deserve to live flourishing lives. But what does a flourishing life look like for Autistic people? We suggest that the hidden biases, methodological errors, and key assumptions of autism science have obscured answers to this question. Here, we seek to initiate a broader discussion about what the foundations for a good Autistic life might be and how this discussion might be framed.

**Recent Findings:**

We identify five ways in which autism science can help us all to secure those foundations, including by (1) giving Autistic well-being prominence in research, (2) amplifying Autistic autonomy, (3) attending better to everyday experiences, (4) acknowledging context, and (5) working in partnership with Autistic people and their families and allies to ensure that they are at the heart of research decision-making.

**Summary:**

Such an approach would direct the focus of autism research to help shape good Autistic lives.

## Introduction

At the core of all good societies is an effort to enable all people to live full and flourishing lives, regardless of their background or characteristics. There has been much debate about the key elements of human flourishing, dating back to at least Aristotle, who wrote of people’s need to “live well and do well” or, in short, to live a good life. Yet, remarkably, the idea of flourishing lives has rarely been applied to Autistic^1^ people. Its absence, we contend, is rooted in the field’s historic commitment to the conventional medical model, in which a person’s functional limitations or “impairments” are the source of any disadvantages experienced, which can be remedied by treatment or cure [1–3]. The absence of any meaningful discussion of flourishing could be because the challenges facing Autistic people can be substantial and lifelong [4••], with the fundamental idea of a “good life” thus seeming out of reach. Or it might be that Autistic people have seldom been invited into the conversation about what such a good life constitutes for them, meaning that conventional accounts of human flourishing seem a long way from any putative Autistic norm. Whatever the reasons, it means that we do not know what a flourishing life might look like for Autistic people nor do we fully understand how we can help to build a foundation for such lives.

Here, our aim is not to provide a full account of Autistic flourishing. That is not our right, nor could it be achieved in a single paper. Rather, we aim here to outline a framework to help Autistic people, their families and allies, researchers, practitioners, and policymakers alike to begin a meaningful conversation about what Autistic flourishing may involve. We suggest that such understanding will require us to step away from key assumptions that have structured autism science for decades now—and to acknowledge the ways in which both hidden biases and methodological errors too often obscure understanding the key elements of Autistic flourishing.^2^ In what follows below, we outline five ways in which we, as an autism research community, need to reconsider Autistic flourishing, functioning almost as a toolkit for those seeking to have conversations about such flourishing well into the future.

## From Health to Well-Being

The first among these is a need to move from a perspective that says the core to Autistic flourishing is simply conventional “physical and mental health” to one that says it is the far broader notion of “well-being” [5]. Autism researchers have largely inherited a medical model that says “physical and mental health” is what matters. They have historically seen autism as a disability in the medical sense: an individual health problem with a biological cause, which requires diagnosing, treating, and fixing [6–9].^3^ As such, autism research often remains firmly embedded within the conventional medical paradigm [10•]—prioritizing a putatively objective standard of “health” over a first-person understanding of “well-being,” which is often dismissed as being overly subjective or biased to personal perception [11]. This priority given to the conventional medical model is evidenced by the way autism science routinely seeks to identify the genetic, neurobiological, and cognitive mechanisms that might explain the highly heterogenous behavioral manifestations of autism [12–15]. Autistic people’s behavioral, cognitive, and neural functionings are therefore frequently compared to some typical or “normal” level of ability, cast as an ideal “state of health.” Interventions and treatments are designed to be applied to the individual to remediate any apparent shortcomings, bringing Autistic people into line with the accepted norm [16, 17].

As should be immediately apparent, this narrow focus on health results in a radically constrained understanding of the value and shape of Autistic lives. It also leads to a much-discussed tendency to see elements of the Autistic experience that stand outside the norm as “deficits,” “impairments,” or “deviations” [10•, 18], independent of what those elements happen to be. Even when Autistic people outperform non-autistic people in scientific experiments, it is sometimes supposed that those achievements reveal a problem with Autistic people that needs to be fixed, rather than simply revealing a different (even potentially “better”) way of being [16, 17, 19, 20]. As we have argued elsewhere [21•, 22••], the medical model, therefore, does not only narrow the range of issues that are given focus, it also “colors” the broader community’s perceptions of Autistic people’s capacities. All of this has sharp real-world implications. It is an approach that is linked to heightened experiences of stigma [23]. It also continues to shape clinical, educational, and public policy responses, with Autistic people being encouraged to fit norms and pursue aspirations apparently demanded by their “health” rather than attending to their wider sense of well-being. If we want to work toward a theory of Autistic flourishing, our contention here is that we need to shift away from this conventional medical paradigm [21•].

The good news is that there are changes afoot already in this regard. The World Health Organization (WHO) has long held that “health is a state of complete physical, mental and social wellbeing and not merely the absence of disease or infirmity” [24] and more recently has recognized “that persons with disabilities have the right to the enjoyment of the highest attainable standard of health without discrimination on the basis of disability” [25]. Consistent with this view, the WHO’s International Classification of Functioning, Disability, and Health (ICF) seeks to provide a comprehensive, integrated framework, recognizing the role of social and environmental factors in the creation of disability, as well as an individual’s health conditions and their effects [26, 27]. Critics may understandably continue to worry that these efforts do not go far enough to shift the emphasis of biomedical views of disability [28, 29], but there is an apparent consensus emerging that a broader range of concerns need to be considered for Autistic people to consider themselves to be leading good lives than is apparent to those constrained by the more conventional medical approach.

## From Other-Defined to Self-Defined

As efforts are made to broaden the range of issues that are taken to be constitutive of a good Autistic life—that is, away from narrow medical concerns to other elements that can meaningfully be considered to be integral to well-being —it is crucial to identify, next, who it is that is empowered to determine what an Autistic good life might be. In the past, Autistic people have been expected to fit a series of requirements laid down by conventional medical practitioners and other experts, who are themselves not Autistic. A properly full account of Autistic flourishing cannot, however, be satisfied with that approach. To capture the fundamental aspects of Autistic life, any attempt to outline the key elements of Autistic flourishing must be primarily determined and defined by Autistic people themselves.

The second essential move is therefore from an “other-defined” framework to a “self-defined” one. At the moment, even when researchers and policymakers use the language of “well-being” and “quality of life” rather than medicalized concerns, these are often derived from a set of standard “life achievements,” irrespective of whether those outcomes are considered meaningful by Autistic adults themselves [30, 31•]. Autistic people repeatedly fare badly on normatively conventional life goals, including friendships, intimate relationships, and workplace success [32–34], and it is often concluded, as such, that Autistic people have a poor quality of life [though see 35, 36]. Of course, many of these normative life achievements are, in fact, important both for Autistic and non-autistic people. But they do not by themselves provide a sufficient account of Autistic well-being nor do they recognize that well-being for Autistic people might differ from well-being for non-autistic people. That is because judgments as to the quality of Autistic lives have all too often been made from outside Autistic experience rather than from within [37••, 38•]. Ne’eman [31•] describes a “disturbing trend” in the way that “researchers, clinicians, and paraprofessionals are using measures that prioritise reducing diagnostic traits that are neither harmful nor personally distressing” to Autistic people themselves (p. 569).

Again, we have begun to see some progress in this regard. There are now a series of studies comparing traditional, researcher-defined objective measures with more subjective measures. What those studies have demonstrated is that outcomes on these measures do not always match up [39–42]. That is, normatively defined “successful” outcomes (independent living, for example) do not always go hand-in-hand with better quality of life, highlighting the importance of both examining the intersection between the person and their environment [42, 43••] and understanding the ways in which Autistic people can flourish on their own terms.

Notwithstanding, it is not just that we are simply more prone to obtaining misleading results and/or misinterpreting these results if we do not attend to subjective perspectives. We fail also to grant Autistic people the dignity, agency, and respect they deserve. It can be taken as fundamental that everyone, including Autistic people, has the opportunity to identify their own desired path in life and, as such, that quality of life should always be measured, at least in part, by the ways in which actual life maps on to the person’s own aspirations.

Another way of putting this is that if we want to take well-being seriously, we need to take Autistic autonomy seriously too [43••, 44•]. As political philosophers Allen et al. [45] explain: “there is no single picture of the flourishing life. What is shared, however… is that human beings are creatures who need to chart their own courses in life. Humans thrive on autonomy, the opportunity for self-creation and self-governance” (p. 18). That is true even of people who are largely dependent on others to help meet their needs [46]. Despite the importance of personal autonomy in allowing people to live a good life on their own terms [47], there has been remarkably little focus on Autistic people’s autonomy [48, 49].

None of this is to say, of course, that Autistic lives are best lived entirely independently or that autonomy is the only value that matters. Achieving full-blown “independence” or self-sufficiency with minimal (or no) support is an impossible standard for anyone [50] and can be a difficult expectation for Autistic people to bear since it denies that interconnectedness shapes everyone’s life [51]. We all depend on others throughout our lives—that is, we are *inter*dependent—and that is, in fact, a good and vital part of life [52–54]. There are, furthermore, always limits on autonomy. Actions that are injurious to others or even those that are profoundly injurious to ourselves are generally understood to be the legitimate subject of external constraint. This was the foundation of the famous “harm principle” in the Victorian philosophy of John Stuart Mill [55], from where the modern concept of autonomy is often drawn. What matters, though, is that each of us is treated as the primary source of our own fundamental judgments and is supported to direct our lives as free from external control as possible.

One reason why this matters so much is that Autistic people, who are non-speaking, have an intellectual disability and/or have high support needs, have sometimes been considered unable to communicate or conceptualize their precise wishes, and, as such, are said to be incapable of being in control, even in part, of their own lives [44•, 56••]. This view is both overly pessimistic and can have damaging consequences—for individuals and communities. There is much to learn from the literature on self-determination in people with intellectual disability. This work clearly demonstrates that those who show more self-determined, agentic behaviors (e.g., choice/decision-making, problem-solving, and self-regulation) also have a greater quality of life [57] and has also identified the many ways in which environments can be created that empower intellectually disabled people to make decisions for themselves [58]. If we want to come to a theory of Autistic flourishing, we need to understand what a good life means to Autistic people themselves, regardless of their support or communication needs, as well as to acknowledge that Autistic lives can indeed be flourishing ones.

## From the Big to the Small

This focus on Autistic autonomy then takes us to our third “building block” of a theory of Autistic flourishing: the move from the big to the small. This may initially seem counter-intuitive. Flourishing, after all, sounds like a “big” idea, a grand theory of what it means to live well. But once we move from an other-defined to a self-defined theory, and begin to ask Autistic people what actually matters in life to them, research reveals that (like most other human beings) they quickly tell us it is not generally the “moonshots” of academic research or public policy-making that excites them, but a range of everyday concerns that build up to be the weft of ordinary life.

This first became apparent to one of us 10 years ago; when together with colleagues, we examined the state of autism research in the UK—how much had been spent on autism research and what it had been spent on [59]. We then asked Autistic people, their families, practitioners, and researchers about their thoughts on the decisions made and where the funds toward autism research should be prioritized in the future. While Autistic people and their families were impressed by the quality of British autism research, they were not at all convinced that it had anything to do with what we now call Autistic flourishing. As one woman said “I fill in all these questionnaires and do everything I can to help… but when it comes down to it, it’s not real life” [60]. Too many people felt that there was a huge gap between the more grand, abstract knowledge that was often produced by research and their real-life, everyday experiences [61]. It fails to tell us, for example, how to help Autistic people catch the bus by themselves, do the groceries, get a date, or complete the paperwork to secure their insurance or social security benefits.

One concrete example of this disconnect between “lab and life” comes from the field of executive function. Researchers have long held that Autistic people have executive problems and that such problems at least superficially map onto reported difficulties in everyday, adaptive behavior [62, 63]. Yet, despite decades of research on executive function in Autistic people, the existing scientific literature—conducted predominantly within carefully controlled lab-based settings where one can manipulate variables and discover causal relationships between one factor and another—has been branded as contradictory and “confusing” [64], with, according to a recent meta-analysis, group differences of only moderate effect [65]. Such effects are a far cry from the large effect sizes reported on (self-/parent-report) questionnaire measures of real-world planning/flexibility difficulties. Indeed, self-reports describe everyday executive issues to be highly variable and depending, critically, on the context in which the task is done (like cooking at home vs. cooking at school), a person's mental state (especially anxiety), the clarity of the task instructions, and the motivation or interest in doing the task [66, 67]. The exclusive reliance on lab-based work, however, has resulted in paradigms that are “simple, contrived and artificial” [68] and fail to achieve representativeness (the correspondence between the task and real-life settings) and generalizability (the degree to which task performance predicts problems in real-life settings) [69]. To put that another way, how can we hope to foster flourishing when the research is decontextualized from the lived reality of Autistic lives?

This issue was also emphasized by participants in a study we conducted examining Autistic people’s experiences during the COVID-19 pandemic [70]. What many Autistic people felt most deeply was the loss of their everyday routines and expected experiences, and they also felt that nobody had noticed how important that was. Owing to the lockdown, participants were unable to engage in everyday activities that many had strived hard to access in the past and which were essential for their well-being—like going to the library, the local swimming pool, the cinema, the mall, sports training or dance class, and the playground with the kids. As one Autistic parent put it, even “the meaningless stuff had become… meaningful and needed” (p. 922). As a research community, we have been too neglectful of the “mundane” aspects of life that matter to people. If we want to work toward an account of Autistic flourishing, then we need to move from the big to the small.

## From the Individual to the “Individual-in-Context”

We have, then, often neglected the broader context which creates the range of real opportunities for Autistic people. That takes us to the fourth element—the need to move from an individual understanding of flourishing to a contextual one. Pervasive adoption of the medical model in conventional autism research has also meant that there is an overemphasis on specific attributes of individuals as opposed to the broader contexts in which Autistic people live [21•]. In the conventional medical view, autism and its associated disabilities are seen as something inherent to the individual. Biomedical research tends not to explain an Autistic person’s difficulties with reference to the context in which the difficulty occurs, but rather as a characteristic of the individual themselves [23]. It follows that the “fault” for difficulties in life resides with the individual themselves—and treatments and interventions are designed in such a way as to “correct” said faults.

The archetypal example of this phenomenon comes from the treatment of Autistic people’s “social deficits.” “Pervasive deficits in social communication and social interaction across multiple contexts” are, according to diagnostic manuals, hallmark features of being Autistic [18]. These difficulties have long been thought to be rooted in impoverishments in the “theory of mind” [71] and/or “diminished social motivation” [72] and subsequently place Autistic people at a significant disadvantage in terms of some traditional life achievements to which we referred earlier—their ability to develop meaningful friendships and intimate relationships and to obtain and sustain work—and even to achieve “normal human experience” [73]. Seen through the lens of the conventional medical model, many treatments for Autistic people, especially young children, have been devised to correct such “deficiencies” [74, 75], helping to “guide brain and behavioural development back toward a normal pathway” [76, p. 776].

In designating these social difficulties as some sort of deficit needing to be fixed, we have neglected to notice that it is generally the broader context which creates the problematic response, not necessarily the behavior itself. By definition, social interactions are shaped by *all* parties—but, until recently, there has been remarkably little focus on the nature of these interactions, especially non-autistic contributions to these interactions. Milton’s [77••] influential double empathy problem has done much to address this concern, emphasizing that there may be a bidirectional misalignment between the minds of Autistic and non-autistic people, resulting in a breakdown in reciprocity and mutual understanding. This, in turn, may be a primary source of social communication difficulties between Autistic and non-autistic people [77••, 78•, 79, 80]. It is surely plausible to suggest that attention would be better directed toward social interventions that aim to shift the negative perceptions of Autistic people’s interaction styles and encourage more relational ways of being [81•]. It is the contextual *response* to the individual that we need to address.

All of this has powerful lessons for what we need to think about. For if the possibilities of flourishing depend not just upon the individual but instead what political philosopher Deva Woodly calls “the individual-in-context” [82], then we need to move our attention away from its sole focus on the individual and appreciate instead what it is that makes anything possible or impossible for them [22••]. That means accepting that the world needs to change, as well as the individual. As Woodly explains: “the context of the individual includes the home, family, community, and nation that they are born into, the physical environment that they exist in, and the structural conditions that link them to socially intelligible categories and political history, therefore organizing the consequences of their being in the world” [82, p. 118]. Without that, our account of flourishing will always remain partial.

## From Researcher-Led to Autistic-Led

These four elements of a theory of Autistic flourishing—moving from health to well-being, from other-defined to self-defined, from the big to the small, and from the individual to the contextual—may initially seem self-evident. Consequently, the fifth element of a theory of Autistic flourishing asks why so many of us in the autism research community missed these first four for so long. In part, we believe this is because of a long history of theory of mind research, which suggested that Autistic people have an impaired ability to reflect on their own mental states [71]. This has led to the questioning of the veracity of Autistic people’s accounts of their own experiences: these are often seen as unreliable [83], and researchers have therefore often avoided attending to first-person testimony, privileging reports from other informants [84], or laboratory-based observation over the perspectives of the person themselves [85••, 86, 87].

As Autistic self-advocate Donna Williams [88] described “Right from the start, from the time someone came up with the word “autism,” the condition has been judged from the outside, by its appearances, and not from the inside according to how it is experienced” (p. 14). As well as shaping research findings, this lack of attention to Autistic people’s perspectives has the consequence of ensuring that Autistic people themselves have almost no say as to what gets researched in autism science, why, or how. So Autistic flourishing has not received attention, and we have missed the elements central to understanding it, because we simply have not asked Autistic people what it is for them to flourish. We have, until recently, assumed that, in order to flourish, Autistic people must need to be less Autistic and that their Autistic identity—their Autistic characteristics, traits, and behaviors—could not possibly contribute to their flourishing since (often) it contradicts or problematizes neuronormative or non-autistic experiences of flourishing.

Ever since the onset of autism research, autism science has typically been designed and conducted without any significant input from Autistic people and their families [89]. It is likely this has contributed toward research agendas and methods that rarely relate to the challenges Autistic people face. And when we say “input,” we mean beyond input as passive participants or subjects in research. What is required, in other words, is input in the decision-making processes around research, in the design and implementation of the research, and the analysis and interpretation of the findings, that is, being partners in the research. And, as we know from our own work, that lack of involvement results in real feelings of disenfranchisement [60, 90•, 91, 92].

Encouragingly, in the last decade, this too has begun to change. There is a slow but growing movement toward collaborating with Autistic people and their allies as part of the research process, where Autistic researchers and community members are actively involved in making decisions about research [93, 94], as well as leading research [95••]. These decisions can include what kind of research is done, how it is done, how research results are interpreted, and how the findings are used. These so-called participatory processes draw on the “practical wisdom” of non-scientists and have been shown outside the field of autism to have a dramatic effect on both the research agenda [96] and on the effectiveness of the research [97]. The idea is that we will learn more, understand more, and know more, once we put lived experience and research experience together.

## Conclusion

Here, we have sought to re-direct the attention of autism researchers to the foundations of Autistic flourishing. Identifying what a flourishing Autistic life looks like should, we argue, be core to our collective endeavors in the years ahead. Getting that process started means recognizing that subjective well-being matters as well as bodily and mental health; stresses that Autistic people should have the same interest in choosing their own lives as anyone else; accepts that the everyday aspects of life matter to people, not just those that seem big from the outside; understands that the context in which people live always shapes opportunities and is therefore an appropriate subject for research; and contends that we will only notice any of this if we work side-by-side with Autistic people and their allies when designing and conducting research. Together, these points are, we contend, the framework for debating Autistic flourishing well and a means of ensuring that autism research can play an active role in helping to shape good Autistic lives.

## Footnotes

1 In the Autistic community, identity-first language (“Autistic person”) is often preferred to and considered less stigmatizing than person-first language (“person with autism”) [98••, 99, 100]. We therefore use identity-first language throughout. We have also chosen to capitalize the word Autistic to indicate a proper adjective, which reflects its status as a robust and valued identity and shared community, similar to the Deaf community.

2 In traditional positivist research paradigms, the researcher attempts to be impartial, minimizing bias in the search for some objective “truth.” Whatever the intent, however, the researcher’s own expertise, experiences, and assumptions undeniably shape the process. EP is a White non-autistic autism researcher and psychologist, committed both to expanding autism science to ensure it examines topics overlooked or systematically ignored but deemed worthy of research by Autistic people themselves and to conduct such research in partnership with Autistic people and their families and allies. MH is a White Autistic autism researcher and an advocate working for and with the Autistic and autism community to nurture Autistic identity and culture and to build Autistic well-being and dignity through education and co-produced research. We are methodological pluralists, believing in the benefits of the social model of disability and a neurodiversity approach to research and practice. The ideas expressed in this piece are the result of many in-depth conversations we have had over the years with each other and with our many collaborators, colleagues and friends, Autistic, and non-autistic.

3 We note here that, with the rise of chronic diseases and evidence-based medicine, the ethical imperative of the medical model has broadened to include the cure, prevention, and management of health and biological disease [101].
